# Editorial: Unravelling human placental (patho-) physiology at the epigenetic and transcriptome level

**DOI:** 10.3389/fcell.2023.1228803

**Published:** 2023-06-23

**Authors:** Athina Samara, Asma Khalil

**Affiliations:** ^1^ Department of Women’s and Children’s Health, Karolinska Institute, Stockholm, Sweden; ^2^ Department of Neonatology, Astrid Lindgren Children’s Hospital, Karolinska University Hospital, Stockholm, Sweden; ^3^ Center for Functional Tissue Reconstruction, University of Oslo, Oslo, Norway; ^4^ Fetal Medicine Unit, St George’s Hospital, St George’s University of London, London, United Kingdom; ^5^ Vascular Biology Research Centre, Molecular and Clinical Sciences Research Institute, St George’s University of London, London, United Kingdom; ^6^ Fetal Medicine Unit, Liverpool Women’s Hospital, University of Liverpool, Liverpool, United Kingdom

**Keywords:** placenta, scRNAseq, epigenetics, transcriptomics, spatial transcriptomics, exosomes

One of the paradoxes of human development lies in its temporary dependence on an extracorporeal organ, the placenta. The placenta is this complex entity that interfaces with the mother, assuming a multitude of structural and functional roles to sustain the fetus throughout its intrauterine existence. Furthermore, the placenta is the master regulator of nutrient and oxygen supply, efficiently removing waste products from the developing fetus. It also provides critical hormonal support and sustains the developing vasculature. Disruption of any of these roles may have an adverse impact on fetal development.

The complex embryology of the placenta translates into an organ composed of distinct cell lineages arising early in development from a single fertilized oocyte. Research has shown that the normal fetal and placental development rely on epigenetic and spatiotemporal processes (Burton and Fowden, 2015). These mechanisms regulate gene expression and cell-to-cell signaling. In order to predict, prevent, diagnose, or treat pregnancy disorders, such as fetal growth restriction, preeclampsia and preterm birth, it is essential that we understand these mechanisms in humans (Bian et al., 2022; Chen et al., 2022; Liao et al., 2022; Wei et al., 2023).

**FIGURE 1:**
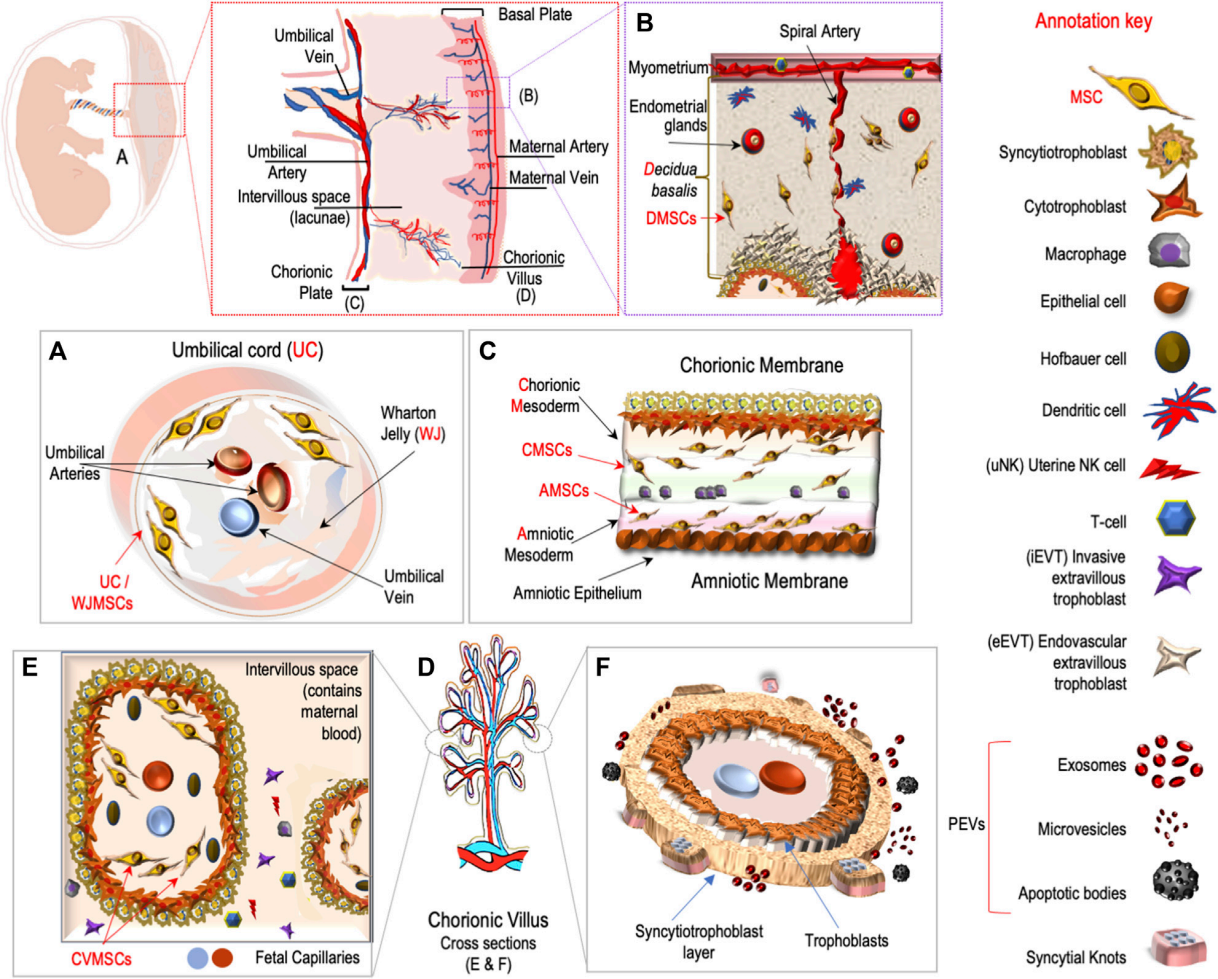
Main placental cell types, sources of mesenchymal stem cells and extracellular vesicles.

In tissues with heterogeneous cell composition and multiple sources of stem cell populations such as the placenta (Figure 1A–C, E), each cell type has a distinct and dynamic epigenetic and transcriptional signature. Thus, the bulk of placental tissue analyses, including bulk sequencing methods, simply profile an amalgamation of the individual signatures of constituent cell types, masking real cell resolution and compromising interpretation. This challenge has been the topic of various studies, with increasing interest and focusing on single cell RNA sequencing to identify the transcriptome and interactions of cells in the placenta (Wächter et al., 2022; Bao et al., 2023; Campbell et al., 2023; Dong et al., 2023; Garcia-Flores et al., 2023).

This Research Topic also aimed to comprehensively address the heterogeneous composition of the placenta in healthy and pathological pregnancies. Liu et al. presented the findings of their investigation into the spatial transcriptome of first trimester human placental villi (Figure 1D, E). They employed a modified spatial transcriptomics approach that was optimized for analyzing loosely structured tissue. One advantage of using spatial transcriptomics in addition to placental single-cell RNA sequencing (scRNA-seq) is the identification of specific cell types within the placental tissue. This enables the mapping and characterization of cell functions, interactions, and the microenvironment in a spatial context, providing valuable insights. Furthermore, the authors emphasized on the use of the modified Stereo-seq method for paraformaldehyde (PFA) fixed samples. This approach allowed them to overcome the challenges associated with collecting placental tissue under special conditions and within a limited time window, which is typically not prioritized. The results demonstrated that PFA fixation significantly enhanced tissue morphology and the specificity of RNA signals compared to using fresh villi embedded in OCT compound.

By employing the spatial transcriptome approach Liu et al. successfully identified the primary cell types present in chorionic villi, including syncytiotrophoblasts, villous cytotrophoblasts, fibroblasts, and extravillous trophoblasts (Figure 1B, E). Additionally, the less abundant cell types, such as Hofbauer cells (Figure 1E) and endothelial cells (Figure 1C), were spatially located through the deconvolution of scRNA-seq dataset. The work of Wang et al. aimed to characterize placental susceptibility to ferroptosis after SARS-CoV-2 placental infection using RNAScope *in situ* hybridization. The technique provided imaging background control combined with less nonspecific hybridization. They further showed that SARS-CoV-2 functioned as a potent trigger for activation of ferroptosis in human placenta, and that the initiation of the placental pathophysiological events resulting from SARS-CoV-2 intraplacental transfer was linked to the pregnancy-related sequelae.

Maternal obesity has been associated with a range of obstetrics outcomes, such as stillbirth, preeclampsia, gestational diabetes, and an increased risk of congenital heart defects in fetuses (Reynolds et al., 2013; Rubens et al., 2022). Obesity during pregnancy significantly contributes to disruptions in these critical metabolic processes, which might trigger endoplasmic reticulum (ER) stress (Burton and Yung, 2011). However, the specific mechanisms linking the obesogenic metabolic environment to adverse pregnancy outcomes remain poorly understood. Shen et al. investigated whether obesity activates the ER stress pathways, also referred to as the unfolded protein response (UPR), in the placenta and evaluated ER stress and UPR activation in the placentas of pregnancies complicated by maternal obesity. Their results revealed that in the obese placenta, p-IRE1α and XBP1s were significantly increased, whereas CHOP and nine UPR chaperone genes were upregulated. By shedding light on these mechanisms, they partially delineated the underlying processes contributing to adverse pregnancy outcomes associated with obesity.

Ortega et al. conducted a timely and comprehensive review that highlights the significance of placental-derived extracellular vesicles (PEVs). The review emphasizes the clinical relevance of EVs (Figure 1F) in various obstetric pathologies, including preeclampsia and gestational diabetes. In recent years, the investigation of changes in PEVs in different obstetric complications has emerged as a promising area of study (Couch et al., 2021). The concentration and cargo of PEVs have been implicated in the development of diverse pathologies, such as preeclampsia, gestational diabetes mellitus, fetal growth restrictions, and placental infections. These changes in PEVs provide valuable insights into the underlying mechanisms of these conditions. The review presents the latest advancements in both basic research and translational aspects regarding the role of PEVs in both normal and pathological pregnancies. The authors advocate for further investigation to explore the potential of PEVs as pathophysiological biomarkers for these diseases. By addressing the importance of PEVs and their association with obstetric complications, this review contributes to the understanding of their clinical significance.

In summary, these studies strongly advocate for the utilization of spatial, bulk, and single-cell analysis methods in future research endeavors to gain a comprehensive understanding of the role and impact of the different ‘omes’ within the placenta. By employing these integrative approaches, researchers can delve deeper into the complexities of placental pathophysiology, uncovering novel insights and potential applications for the exploration of diagnostic or prognostic biomarkers related to various pregnancy disorders. These findings highlight the significance of continued investigation in this field to advance our understanding and improve clinical management of pregnancy-related conditions.
